# Beyond M1 M2: single-cell insights into how glioblastoma reshapes tumour-associated macrophages

**DOI:** 10.3389/fimmu.2026.1930848

**Published:** 2026-10-02

**Authors:** I. Serangeli, L. Ghione, F. Toma, A. Sgambato, D. Lucchetti

**Affiliations:** 1 Department of Translational Medicine and Surgery, Università Cattolica del Sacro Cuore, Rome, Italy; 2 Fondazione Policlinico Universitario A. Gemelli, Istituto di Ricovero e Cura a Carattere Scientifico (IRCCS), Rome, Italy; 3 Multiplex Spatial Profiling Facility, Fondazione Policlinico Universitario A. Gemelli, Istituto di Ricovero e Cura a Carattere Scientifico (IRCCS), Rome, Italy

**Keywords:** glioblastoma, tumour-associated macrophages, microglia, single-cell RNA sequencing, spatial transcriptomics, myeloid reprogramming, lipid metabolism, immunosuppression

## Abstract

Glioblastoma (GBM) is an immunologically cold tumour in which glioma-associated microglia and macrophages constitute the predominant immune cell population, comprising brain-resident tumour-associated microglia (Mg) and infiltrating monocyte-derived (Mo) tumour-associated macrophages (TAMs). Recent single-cell and spatial transcriptomic studies have fundamentally revised the traditional M1/M2 activation paradigm, revealing a dynamic continuum of niche-dependent myeloid states rather than discrete polarisation phenotypes. In this review, we discuss how tumour-derived signals reshape Mg and Mo-TAMs through coordinated transcriptional, metabolic, and spatial reprogramming, with particular emphasis on interferon-responsive and antigen-presenting cell (APC)-like microglia, lipid-laden Mo-TAMs, and immunosuppressive myeloid populations. We critically compare myeloid states identified across independent mouse-derived and human GBM datasets, highlighting both convergent programmes and unresolved discrepancies in how axes such as interferon response, hypoxia and lipid handling are resolved at different taxonomic resolutions across studies. We further examine how metabolic constraints, including lipid accumulation, hypoxia and tryptophan catabolism, together with receptor-mediated inhibitory pathways and cytokine circuits, uncouple transcriptional identity from functional immune competence, creating a central paradox of GBM myeloid biology. Finally, we discuss emerging therapeutic strategies aimed at reversing tumour-driven myeloid reprogramming, including modulation of immunosuppressive circuits, disruption of metabolic symbiosis, inhibition of extracellular vesicles (EVs)-mediated communication, and spatially informed combinatorial immunotherapies. Collectively, we argue that while single-cell and spatial profiling have substantially redefined Mg and Mo-TAM biology in GBM, reconciling mouse-derived and human-derived myeloid taxonomies remains an open challenge with direct implications for translating myeloid-directed immunotherapeutic strategies from preclinical models to patients.

## Introduction

Glioblastomas (GBMs) are typically immunologically cold tumours in which resident microglia and infiltrating monocyte-derived macrophages, constitute the predominant leukocyte population, whereas T-cell infiltration is limited and functionally constrained (1). Within this context, brain-resident tumour-associated microglia (Mg) and infiltrating monocyte-derived tumour-associated macrophages (Mo-TAMs) become a key factor in the immune response to the tumour. Rather than undergoing the canonical polarisation, microglia and monocyte-derived macrophages in GBM are actively reshaped by tumour-driven cues into non-canonical states. Single-cell and spatial analyses indicate that microglia and monocyte-derived macrophages in GBM are educated to transition towards heterogeneous phenotypic programs and that peripheral myeloid cell infiltration is enriched at blood-brain barrier (BBB) disruption areas (2). At the tissue level, myeloid composition and state are spatially patterned, with microglia enriched in interferon/pro-inflammatory signals at the invasive margins, and monocyte-derived macrophages enriched in hypoxic necrotic core regions (3). Schmassmann et al. (4) recently observed a contrasting trend. Their analysis revealed a downregulation of interferon-response genes (such as IFI6, STAT1, and ISG15) specifically in microglia from the periphery compared to the centre, suggesting that the tumour core may harbour a more immunologically responsive microglial pool, potentially stimulated by infiltrating CD8^+^ T cells (4). These studies support that tumour-associated microglia and macrophages are reprogrammed by the tumour into hybrid states, which becomes evident only with high-resolution single-cell and spatial approaches that resolve distinct, plastic programs at the level of individual cells. Microglia and macrophages were originally divided into M1 (“classically activated”), and M2 (“alternatively activated”) based on *in vitro* experiments (5). More recently, this nomenclature evolved into M1-like, typically induced by TNF-α and IFN-γ and associated with pro-inflammatory response, and M2-like, commonly induced by interleukin IL-4/IL-13 and associated with immunoregulatory and tissue-repair programs. This nomenclature, although still widely used, fails to capture the *in vivo* diversity of microglia and macrophages states observed in tumours, especially in GBM (6, 7). This limitation is now widely recognised at the field level: a 2026 international consensus statement on microglial and macrophage functions in gliomas explicitly recommends moving away from simplistic M1/M2 terminology (8). In almost all tumour types, including GBM, single-cell profiling studies have shown that myeloid cells often co-express both M1 and M2 transcriptional programs, not segregating into M1/M2 discrete phenotypes (6, 9, 10). Central to this framework is a paradox that M1/M2 models cannot capture: GBM-associated microglia and monocyte-derived macrophages can acquire transcriptional competence for antigen presentation, yet the same niche that permits this acquisition simultaneously enforces suppressive mechanisms that prevent its functionality, a duality with direct consequences for therapeutics (2, 11). We term this recurring tension transcriptional–functional uncoupling and return to it at each stage of this review as new molecular and spatial evidence refines its mechanistic basis. Pioneering single-cell studies by Klemm et al. (13) and Friebel et al. (12) first established the tumour- and tissue-specific myeloid landscapes of IDH-mutant versus IDH-wildtype gliomas, revealing profound differences in microglial and monocyte-derived compartments across entities and anatomical sites. Building on this foundation, more recent spatial and single-cell datasets in glioblastoma have shifted the focus from binary M1/M2 activation towards niche-dependent myeloid state continua (12, 13). Several recent reviews have discussed the limitations of the M1/M2 paradigm and the emerging complexity of microglia and monocyte-derived macrophages states in gliomas (7, 14, 15). Rather than proposing a new classification per se, our aim is to systematically align multiple single-cell and spatial datasets (2, 4, 12, 13, 16–19) within a single conceptual backbone, highlighting convergent and divergent myeloid states and explicitly flagging unresolved conflicts between studies. This conceptual shift from binary polarization to dynamic state transitions raises a fundamental question: how do homeostatic microglia become reprogrammed into the diverse states observed in GBM? To address this question, we first outline the molecular identity of homeostasis before examining how GBM remodels these cells in a spatially organized manner, giving rise to distinct transcriptional states, including IFN-responsive and APC-like populations. We subsequently examine the metabolic and immunosuppressive mechanisms that constrain these states and conclude by discussing the therapeutic implications of this emerging framework.

## Technical considerations

Before discussing individual myeloid states, we summarize key technical considerations that affect the interpretation of single-cell and spatial datasets in GBM, including platform-specific resolution, batch effects, dissociation artefacts, doublets, and deconvolution biases. These interpretations should be considered in light of known technical limitations of scRNA-seq and spatial transcriptomics (20–24). In scRNA-seq, tissue dissociation can introduce transcriptional artifacts, including stress-response programs (21), while batch effects and dropout can distort clustering, marker detection, and the inferred abundance of rare or fragile populations (23, 24). This is particularly relevant for myeloid subsets such as neutrophils or PMN-like cells, which are especially vulnerable to loss during dissociation and preprocessing (21). Spatial transcriptomics partly preserves tissue context, but most sequencing-based platforms still lack true single-cell resolution and therefore depend on computational deconvolution, which introduces additional uncertainty when assigning cell states *in situ* (20). Spatial transcriptomics platforms used in GBM studies fall broadly into two categories: sequencing-based approaches such as Visium and Slide-seq, which capture multi-cellular spots and therefore require computational deconvolution to infer cell-type composition; and imaging-based approaches such as Xenium, MERFISH, and CosMx, which achieve near-single-cell resolution at the expense of more limited gene panels. As a result, niche assignments and myeloid state localization derived from sequencing-based platforms are more sensitive to deconvolution assumptions, whereas imaging-based platforms provide higher spatial precision but may miss rare or transcriptionally subtle states not included in the panel. States defined by non-myeloid lineage markers, such as astrocytic-like (GFAP^+^) and endothelial-like (PECAM1^+^/CLDN5^+^) microglia-derived tumour-associated macrophages (Mg-TAM), raise the possibility of doublets or engulfment artefacts. In dissociation-based single-cell datasets, microglia–astrocyte or microglia–endothelial doublets could mimic genuine lineage hybridization. Immunohistochemical co-staining (GFAP^+^/Iba1^+^, CD31^+^/Iba1^+^) partially mitigates this concern, yet the doublet hypothesis and phagocytic uptake of lineage-specific material should be explicitly considered when interpreting these states. Accordingly, the myeloid hierarchies inferred from these datasets should be viewed as biologically informative models that benefit from orthogonal validation by flow cytometry, immunohistochemistry, multiplex imaging, and functional assays.

## Homeostatic microglia

At homeostasis, microglia operate as active sentinels of the CNS parenchyma, continuously surveying the local environment and contributing to circuit maintenance through complement-dependent synaptic pruning (25). This baseline identity is not a passive default but an actively maintained programme, sustained by niche-derived signals including TGF-β and neuron-microglia communication pathways, and reliably tracked by the microglia-enriched markers TMEM119 and P2RY12 (26). The regulated and reversible nature of this homeostatic state is underscored by disease contexts in which P2RY12 and TMEM119 are progressively lost upon inflammatory challenge and recover upon its resolution (27). Within the CL0–CL8 framework proposed by Yabo et al. (2), shown in **Figure 1**, CL0 represents the core homeostatic (Ho-Mg) state, characterized by the highest expression of canonical microglial markers (P2RY12, TMEM119 and GPR34) together with the maintenance of the MAF-, NR3C1- and SOX4-centred transcriptional regulons. Functionally, CL0 corresponds to the baseline surveillance phenotype of resident microglia. CL1 retains these core homeostatic regulons but displays reduced expression of the same canonical markers, indicating an attenuation, rather than a loss, of the homeostatic programme. Spatially, CL1 is enriched at the tumour margin within neural development-associated niches, adjacent to OPC-like and NPC-like malignant cell states, as defined by the four-state transcriptional classification of GBM (28). Rather than representing a fully activated state, CL1 appears to define an early transitional stage along the continuum from Ho-Mg to microglia-derived tumour-associated macrophages (Mg-TAMs), consistent with the progressive nature of tumour-induced microglial reprogramming described below. This intrinsic plasticity, the capacity to disengage from the homeostatic programme in response to niche cues, and to re-engage when those cues are removed, provides the molecular substrate for tumour-driven reprogramming in GBM (2). Throughout this review, we treat the CL0–CL8 framework proposed by Yabo et al. as a working scaffold rather than a settled taxonomy. It remains the most granular model of microglial state transitions in GBM currently available, in the absence of an independently re-clustered human taxonomy of comparable resolution, large human references such as GBmap have so far been used to validate or project this mouse-derived framework rather than to generate one of their own. Nonetheless, as detailed below, at least one of its constituent states, CL3, may itself represent a composite population rather than a single, homogeneous transcriptional state once cross-referenced against independent human datasets such as those of Tang et al. (16). We flag this tension explicitly rather than resolve it, since doing so would require exactly the kind of independent human re-clustering discussed above.

**Figure 1:**
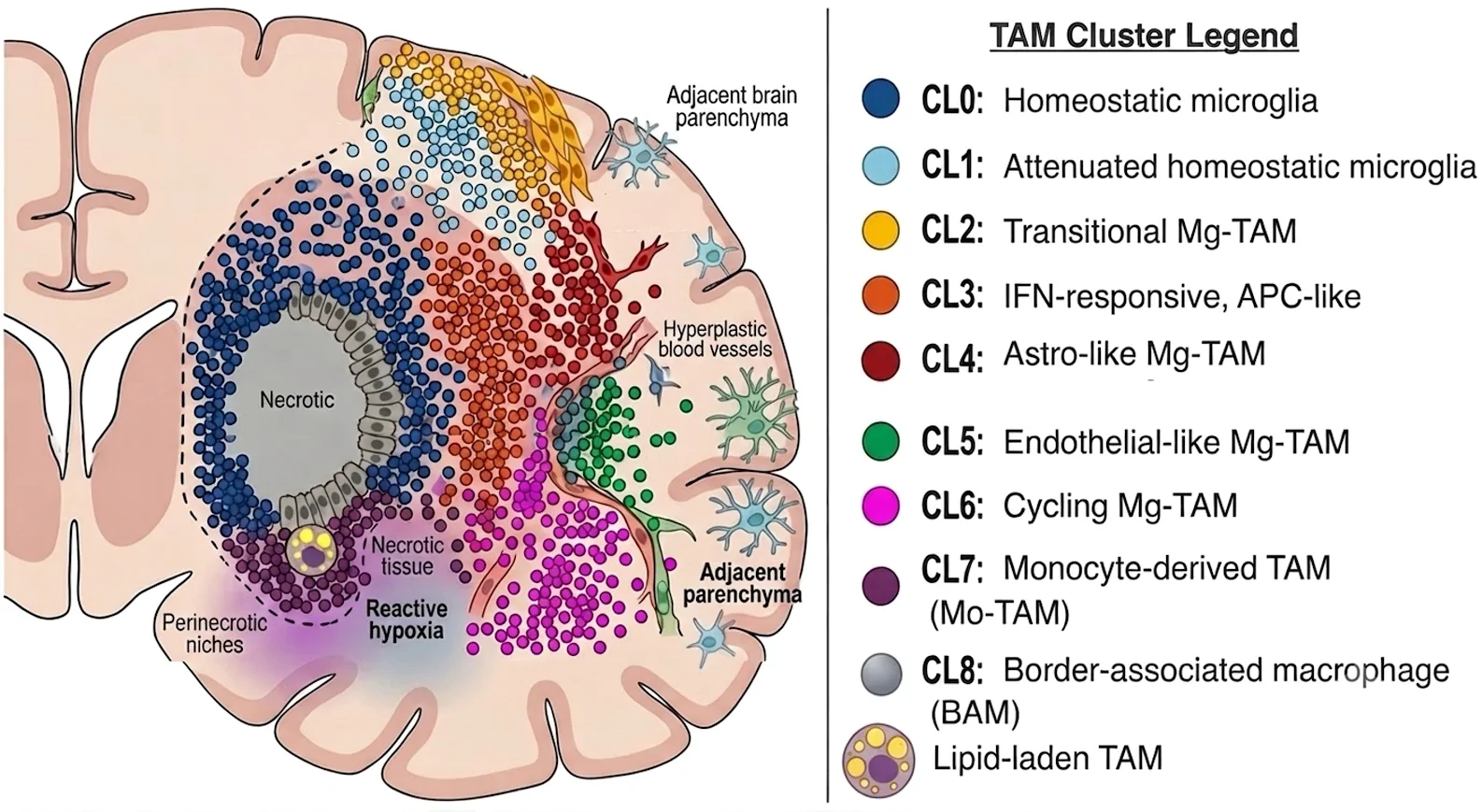
Spatial architecture of the Mg-TAM and Mo-TAM landscape in GBM. Regional distribution of microglia-derived (CL0–CL6) and monocyte-derived (CL7) tumour-associated macrophage states, together with border-associated macrophages (CL8), across the tumour core, perinecrotic/hypoxic regions, perivascular niches, and the infiltrative margin, based on the CL0–CL8 framework (2). Lipid-laden TAMs (32) are shown as a functionally distinct population and are not part of the CL0–CL8 numbering. Spatial assignment is well supported for CL0–CL3; for CL4–CL6 and CL8, niche localization is based on bulk Ivy-GAP correlation and has not been resolved at single-cell or spot resolution in patients (2).

## Tumour-driven microglial reprogramming

Single-cell and spatial transcriptomic studies have demonstrated that GBM-associated microglia do not segregate into discrete M1 or M2 activation states but instead occupy a continuum of transcriptional programmes shaped by spatial niche, metabolic constraints, and tumour genotype (2, 14, 15, 29) as summarized in **Figures 1**, **2**. As displayed in **Figure 1** and **Table 1**, using an integrated high-resolution single-cell and spatial framework, Yabo et al. identified nine microglial clusters in patient-derived orthotopic xenograft (PDOX) models, comprising two Ho-Mg states (CL0–CL1) and five Mg-TAM states (CL2–CL6), one Mo-TAMs state (CL7) and one border-associated macrophage (CL8), throughout the manuscript we therefore avoid using ‘TAMs’ as a generic umbrella term and explicitly state whether we refer to Mg, Mg-TAMs or Mo-TAMs.

**Figure 2:**
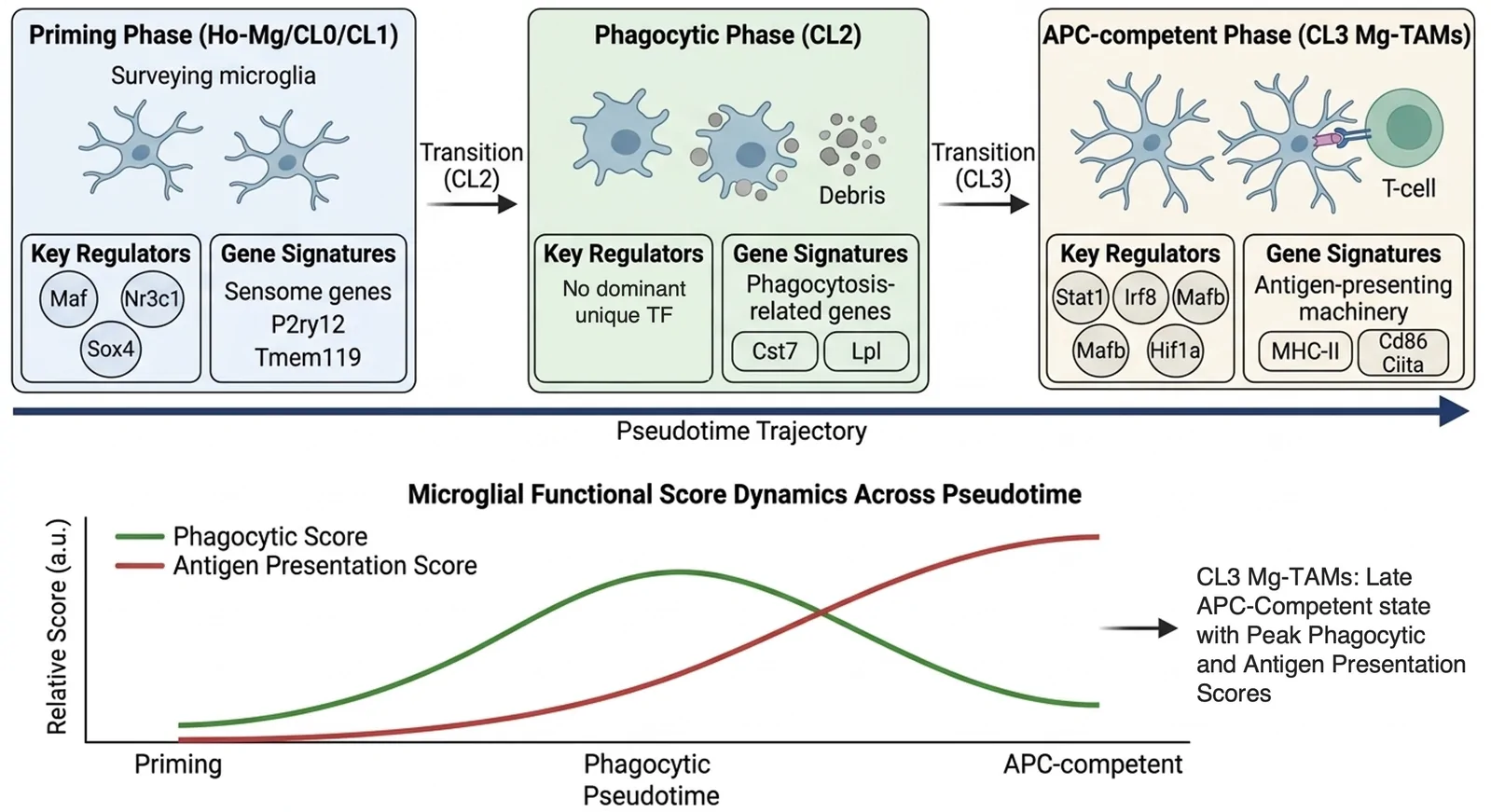
Pseudotime trajectory inference of microglial activation toward the CL3 Mg-TAM state. Schematic reconstruction of the sequential activation model proposed by Yabo et al. (2), progressing from a homeostatic priming phase (CL0/CL1; regulons Maf, Nr3c1, Sox4), through a phagocytic phase (CL2; markers Trem2, Axl, Apoe, Cst7, Lpl — no dominant SCENIC regulon identified for this transitional state), to an APC-competent phase (CL3 Mg-TAMs; regulons Stat1, Irf8, Mafb, Hif1a; antigen-presentation machinery MHC-II, Cd86, Ciita). The lower panel is a schematic, qualitative representation of the reported temporal offset between phagocytic and antigen-presentation functional scores and does not reproduce quantitative data from the original publication (2).

**Table 1:** Microglia-derived states in GBM: the Yabo et al. (2) CL0–CL8 taxonomy.

State/Cluster	Gene signature/markers	Key TFs (SCENIC)	Spatial niche	Functional notes/mechanism	Human correspondence	Reference
Homeostatic microglia (Ho-Mg)						
CL0 (Ho-Mg)	P2ry12, Tmem119, Gpr34 (high homeostatic score)	Maf, Nr3c1, Sox4	Detected across several spatial GBM niches; not restricted to one niche	Baseline surveillance phenotype; highest homeostatic gene expression of all clusters; a subset also expresses dissociation-induced genes (Egr1, Fos), controlled for as a technical artefact rather than a disease state.	Corresponds to resident/homeostatic human microglia expressing P2RY12, TMEM119 and related homeostatic signatures. Partial conceptual overlap exists with the human TAM-AIF1 subset, although TAM-AIF1 additionally displays MHC-II-associated immunomodulatory activity and should not be considered equivalent to CL0.	Yabo et al., 2024 (2)
CL1 (Ho-Mg)	Lower expression of P2ry12, Tmem119, Gpr34 vs CL0	Shares Ho-Mg regulons with CL0 (Maf, Nr3c1, Sox4)	‘Neural development’ niche at the tumour edge, close to OPC-/NPC-like (stem-like) GBM states	Partial loss of homeostatic identity represents an early step along the activation continuum toward Mg-TAM states.	Corresponds to an attenuated resident/homeostatic microglial programme in human GBM, with reduced expression of P2RY12 and TMEM119. It may partially overlap with the human TAM-AIF1 state, but a distinct human counterpart has not been independently resolved.	Yabo et al., 2024 (2)
Microglia-derived tumour-associated macrophages (Mg-TAMs)						
CL2 (Mg-TAM)	Sensome: Cd74, Cd52, Cxcl16, Clec7a (shared with CL3); Classical pro-tumorigenic: Spp1, Cst7, Cxcl13, Apoe (highest in CL2/CL3)	No dominant unique TF; transitory regulon profile between Ho-Mg and CL3	Co-localises with sensome/phagocytosis/antigen-presentation signatures across several niches (bridges Ho-Mg and CL3 along TSCAN pseudotime); not restricted to a single niche the way CL3 is	Turns on the activated sensome programme while homeostatic markers begin to decline; represents the entry point into the Mg-TAM trajectory.	Corresponds most closely to a transitional human myeloid programme bridging resident microglia and activated Mg-TAMs. No independent human Mg-TAM cluster precisely matching CL2 has been established. Pombo Antunes et al. describe an analogous transitional programme within the Mo-TAM (not Mg-TAM) compartment, but this reflects convergent nomenclature across distinct ontogenetic lineages rather than a validated cross-lineage correspondence	Yabo et al., 2024 (2)
CL3 (Mg-TAM)	Sensome: Cd74, Cd52, Cxcl16, Clec7a; Classical: Spp1, Cst7, Cxcl13, Apoe; Secretory: Ccl3, Ccl4 (higher than CL2); High Ptprc (CD45); low homeostatic genes	Hif1a, Stat1, Mafb, Irf8	‘Reactive immune’ niche: high glial/inflammation-associated genes, co-localised with non-hypoxic MES-like GBM states	Most differentiated pro-tumorigenic state; highest phagocytic and antigen-presentation activity and strongest secretory/cytokine output; integrates hypoxia (Hif1a) and interferon (Stat1, Irf8) programmes into a consolidated identity.	Its SPP1/APOE and CCL3/CCL4 components overlap with the human TAM-SPP1 and TAM-CCL3 subsets, respectively, whereas its MHC-II/APC-associated programme overlaps with human HLA-DRA, HLA-DPA1, CD74 and interferon/APC-like states. CL3 should be regarded as a composite mouse-derived state rather than as a single validated human counterpart. Its co-localisation with MES-like tumour regions is also consistent with, but does not establish equivalence to, the human HMOX1^+^CD163^+^ myeloid population described by Ravi et al.	Yabo et al., 2024 (2)
CL4 (Mg-TAM)	Sparcl1, Gfap; low sensome score	Thra, Sox9, Sox2	High signal in leading-edge/infiltrating/cellular-tumour niches (Ivy GAP bulk RNA-seq)	Acquires astrocytic-like transcriptional features (confirmed by GFAP^+^/Iba1^+^ IHC); pro-tumorigenic markers present but weaker/more variable than CL3, possibly reflecting phagocytosis of astrocytic material. Spatial enrichment is bulk-level (Ivy GAP) and not resolved at single-cell/spot resolution.	Human analogue plausible but not robustly resolved in patient single-cell data.	Yabo et al., 2024 (2)
CL5 (Mg-TAM)	Pecam1, Cldn5; low sensome score	Not uniquely defined in source; shares broader Mg-TAM/Mo/BAM regulons	High signal in hyperplastic-blood-vessel (HyBV) and microvascular-proliferation (MvP) niches (Ivy GAP bulk RNA-seq)	Acquires endothelial-like transcriptional features (confirmed by CD31^+^/Iba1^+^ IHC), possibly reflecting phagocytosis of endothelial material. Spatial enrichment is bulk-level (Ivy GAP) and not resolved at single-cell/spot resolution.	Human analogue plausible but not robustly resolved in patient single-cell data.	Yabo et al., 2024 (2)
CL6 (Mg-TAM)	Top2a, Mki67; low sensome score	Tfdp1, Atf1 (classical cell-cycle regulators)	High signal in cellular-tumour, HyBV and MvP niches (Ivy GAP bulk RNA-seq)	Active cell-cycle programme (confirmed by Ki67^+^/Iba1^+^ IHC); a distinct proliferative Mg-TAM state. Spatial enrichment is bulk-level (Ivy GAP) and not resolved at single-cell/spot resolution.	Human analogue plausible but not robustly resolved in patient single-cell data.	Yabo et al., 2024 (2)
Peripherally derived TAMs						
CL7 (Mo-TAM)	Pro-tumorigenic: Cxcl13, Ctsd, Ccl4, Apoe; Mg-mimicry: Spp1, Trem2, Cst7, Tyrobp	Fosl2, Irf1, Cebpb	‘Reactive hypoxia’ niche (spatial transcriptomics); also, HyBV/MvP/perinecrotic/pseudopalisading areas (Ivy GAP bulk), all sites of BBB leakage	Distinct ontogeny from resident microglia; monocytes transition toward TAM phenotypes with partial Mg mimicry, restricted to BBB-compromised, hypoxic/necrotic niches.	Corresponds to human infiltrating monocyte-derived TAMs, particularly the hypoxic and reactive monocyte-derived programmes identified by Pombo Antunes et al. Partial overlap may also exist with their phagocytic/lipid, SEPP1^+^ and interferon-signature subsets, but no single human population recapitulates the complete CL7 signature.	Yabo et al., 2024 (2)
CL8 (BAM-TAM)	Upregulates Mg gene Clec7a; downregulates BAM markers Cd163, F13a1, Mrc1	Bach1, Prdm1, Klf4 (shared with Mo)	Shares BBB-leakage niches with Mo (HyBV, MvP, perinecrotic, pseudopalisading; Ivy GAP bulk); not separately resolved in spatial transcriptomics due to low cell numbers	Transcriptional convergence toward a microglia-like identity while losing classical BAM markers, reflecting local GBM instruction.	Human analogue plausible but not robustly resolved in patient single-cell data.	Yabo et al., 2024 (2)

Gene signatures, transcription factors, spatial niches and human correspondence for microglia-derived (Mg-TAM) and monocyte/BAM-derived (Mo-TAM, BAM-TAM) states identified by Yabo et al. (2) in murine PDOX and GL261 models, with human relevance established via signature projection.

As shown in **Table 1**, it is important to note that the CL0–CL8 clustering was derived exclusively from mouse-derived myeloid cells (PDOX and GL261 Drop-seq data), and not from human GBM cells directly. Large-scale harmonized human single-cell references, such as GBmap, which integrates over 1.1 million cells from 240 IDH-wildtype GBM patients (30), represent a critical resource against which the CL0–CL8 taxonomy, and in particular the pro-tumorigenic CL3 state, should be independently validated in future studies. Human relevance was subsequently established via gene-signature projection onto independent human scRNA-seq datasets (14) and spatial transcriptomic mapping (31), rather than through independent re-clustering of human data. Correspondence is strongest for CL0–CL3, whereas CL4–CL8 rely primarily on bulk spatial correlation and remain less resolved at single-cell/spot resolution in patients, as acknowledged by the original authors (2), a caveat that applies throughout this review whenever CL4–CL8 are discussed. Complementary high-depth regional scRNA-seq analyses of matched tumour core, infiltration zone, and peripheral blood further confirmed this transcriptional heterogeneity, demonstrating that resident microglia and infiltrating monocyte-derived macrophages acquire spatially distinct activation programmes that cannot be captured by bulk or single-region analyses (2, 4).

Yabo et al. find by trajectory inference analyses that these microglial populations represent sequential stages along a shared activation continuum rather than distinct cellular identities. Within this framework, CL2 represents a transitional state bridging Ho-Mg and activated Mg-TAMs, whereas CL3 displays the most differentiated pro-tumorigenic phenotype. Additional specialised Mg-TAM states further illustrate the remarkable plasticity of resident microglia. Astrocytic-like Mg-TAMs (CL4) express astrocytic markers, including GFAP and SPARCL1, likely reflecting the phagocytosis of astrocytic material at the invasive tumour margin. Endothelial-like Mg-TAMs (CL5), characterised by the expression of PECAM1 and CLDN5, preferentially localise to perivascular niches, whereas cycling Mg-TAMs (CL6), defined by proliferation-associated genes such as MKI67 and TOP2A, account for the local expansion of the microglial compartment (2).

Independent single-cell studies have further highlighted the functional heterogeneity of TAMs across glioma grades. Using scRNA-seq of grade 2 and 3 glioma samples, Tang et al. identified three principal TAM populations with distinct biological and clinical associations and subsequently validated a derived four-gene prognostic signature in a bulk TCGA cohort spanning WHO grades 1–4 (**Table 2**) (16); direct single-cell characterisation of these subsets in grade 4/GBM tissue is therefore not yet available, and their relevance to GBM specifically should be regarded as inferred rather than directly demonstrated. Firstly, the TAM-AIF1 subset retains Ho-Mg markers, including AIF1, P2RY12, and TMEM119, while exhibiting MHC-II-mediated immunomodulatory activity that contributes to T-cell exhaustion (16). Secondly, the TAM-SPP1 (lipid-laden) subset is enriched for SPP1, APOC1, APOC2, and TREM2, suppresses T-cell activation through the SPP1–CD44 and LGALS9–CD44 signalling axes, and is associated with EGFR amplification and poor patient survival (16, 17, 32). Independent human evidence further supports a pro-tumorigenic, therapeutically targetable role for SPP1^+^ macrophage-like populations: in GBM, SPP1^+^ monocyte-derived macrophages preferentially accumulate in anti-PD-1 non-responders via a tumour-intrinsic SDHAF2–ITGA5 axis, and antibody-mediated ITGA5 blockade restores anti-PD-1 efficacy (33). Thirdly, the TAM-CCL3 population, characterised by high expression of CCL3, CCL4, and IL1B, is uniquely associated with improved clinical outcome, suggesting a potential anti-tumour function. These TAMs states are shown in **Table 1**. Note that in Yabo’s mouse-derived Mg-TAM CL3, the SPP1/APOE program and CCL3/CCL4 secretory program co-occur within the same cluster, whereas Tang et al.’s independent clustering of human tissue resolves these into two populations with opposite prognostic associations (TAM-CCL3: favourable; TAM-SPP1: unfavourable) (16). This raises the possibility that CL3, as defined in the mouse/PDOX system, aggregates transcriptional programs that are functionally and prognostically separable in human glioma tissue, a discrepancy that limits direct translation of CL3-targeted therapeutic rationale without further resolution in human single-cell data.

**Table 2:** Human TAM subsets (16), identified independently of the CL0–CL8 framework.

State/Cluster	Gene signature/markers	Key TFs (SCENIC)	Spatial niche	Functional notes/mechanism	Human correspondence	Reference
TAM-SPP1 (“lipid-laden”)	SPP1, APOC1, APOC2, TREM2	SREBF1 (drives lipid metabolism/OXPHOS programme)	Associated with EGFR-amplified tumours; identified by scRNA-seq in grade 2–3 glioma; prognostic signature validated in bulk TCGA (WHO grades 1–4)	Suppresses T-cell activation and promotes T-cell exhaustion via the SPP1-CD44 and LGALS9-CD44 axes; associated with poor patient survival.	Human TAM subset identified independently of the Yabo CL0–CL8 taxonomy.	Tang et al., 2024 (16)
TAM-CCL3 (“activated/pro-inflammatory”)	CCL3, CCL4, IL1B, CCL3L1, CCL4L2	Bcl3, Nfkb2 (also shares Mef2c with TAM-AIF1)	—	Uniquely associated with improved patient survival among the three Tang subsets, opposite direction to TAM-SPP1/TAM-AIF1. Conceptual overlap with Yabo CL3: Ccl3/Ccl4 are part of Yabo’s CL3 secretory signature, where they mark the most pro-tumorigenic Mg-TAM state. Tang’s TAM-CCL3, built on largely the same cytokine genes, is instead protective. Same markers, opposite functional read-out, do not treat as the same population.	Human TAM subset identified independently of the Yabo CL0–CL8 taxonomy.	Tang et al., 2024 (16)
TAM-AIF1 (“resident-like”)	AIF1, C1QA, C1QB, HLA-DRB1, P2RY12, TMEM119	Mef2c (shared with TAM-CCL3)	—	Retains homeostatic/resident features but drives T-cell exhaustion through MHC-II-mediated interactions. Conceptual overlap with Yabo CL0/CL1: P2ry12 and Tmem119 are the same core homeostatic markers Yabo uses to define Ho-Mg. This is the closest cross-study correspondence in the table, though Tang additionally reports an immunosuppressive MHC-II function for this state that Yabo does not attribute to CL0/CL1.	Human TAM subset identified independently of the Yabo CL0–CL8 taxonomy.	Tang et al., 2024 (16)

These three subsets were identified by independent scRNA-seq clustering of human grade 2–3 glioma tissue (not GBM/grade 4 directly) and validated in a bulk TCGA cohort spanning WHO grades 1–4. They are presented separately from **Table 1** because they derive from a distinct analytical framework and, in the case of TAM-CCL3, show a prognostic association opposite to the superficially similar Yabo CL3 secretory programme (see main text for discussion).

Independent, cross-species evidence for niche-dependent myeloid heterogeneity in GBM was provided earlier by Pombo Antunes et al. (14), who combined scRNA-seq and CITE-seq across mouse GL261 tumours and human GBM at both newly diagnosed and recurrent stages (14). This study established, ahead of and independently from the PDOX-based framework of Yabo et al. (2), that microglia-derived and monocyte-derived TAMs constitute self-renewing populations that compete for anatomical niches and are differentially depleted by CSF1R blockade; microglia-derived TAMs predominate at diagnosis but are progressively outnumbered by monocyte-derived TAMs at recurrence, particularly within hypoxic tumour regions. Within the monocyte-derived compartment, the authors resolved five conserved subsets, transitory, phagocytic/lipid, hypoxic, SEPP1^+^, and interferon-signature, validated by direct differential-expression comparison between human and mouse data rather than by signature projection alone. Notably, the hypoxic and interferon-signature programs segregated into distinct clusters in this independent analysis, in contrast to their integration within a single population (CL3) in the mouse-derived Yabo taxonomy, suggesting that the resolution at which these two axes are separated may depend on the reference dataset and clustering strategy employed (14).

Taken together, these cross-study comparisons suggest that the CL0–CL8 framework provides a coherent model of microglial activation in GBM. However, it has not yet been fully reconciled with independent human and cross-species datasets at the level of individual Mg-TAM states. Across studies, human-based clustering consistently identifies distinct programmes that are grouped into a single population (CL3) in the mouse-derived taxonomy. In the study by Tang et al., these programmes even show opposite prognostic associations. This suggests that CL3 may represent a composite population rather than a single, homogeneous transcriptional state. This distinction is more than semantic. It has important implications for the therapeutic strategies discussed later, which are largely based on CL3 biology. It also highlights the need for higher-resolution human single-cell and spatial datasets before findings from the PDOX model can be confidently translated to patients. Concretely, a therapeutic strategy designed to reprogram or deplete CL3 Mg-TAMs as a monolithic target risk simultaneously affecting a favourable-prognosis CCL3/CCL4-driven programme and an unfavourable-prognosis SPP1-driven programme, potentially blunting efficacy or producing unpredictable clinical outcomes until these programmes can be resolved as distinct targets in human data. With this limitation in mind, the next section focuses on the CL3 Mg-TAM state, as it remains the best-characterised and most functionally informative population within the current framework.

## CL3 Mg-TAMs: IFN-responsive and APC-like states

Among the microglial clusters identified in GBM, CL3 Mg-TAM (2) emerges as the most differentiated pro-tumorigenic state, integrating a Stat1-driven interferon response with professional antigen-presenting cell (APC) features (as described in detail in **Table 1**). While human interferon-stimulated genes (ISGs) like IFI6, IFI27, and MX1 canonically define IFN-responsive TAM states in human datasets (29), Yabo et al. (2) defined the mouse CL3 state through an activated sensome programme (CD74, CLEC7A) and a potent antigen-presentation machinery, including MHC-II molecules (H2-Ab1, H2-Eb1), CD86, and the master regulator Ciita. Transcription factor analysis indicates that a Stat1-driven regulon dominates this “APC-competent” CL3 Mg-TAM state. This finding is significant because it suggests that the dendritic-like programme observed in GBM-associated microglia is a linear progression of the interferon (IFN)-responsive state rather than a distinct, independent polarisation event. As shown in **Figure 2**, pseudotime reconstruction supports a sequential activation model in which microglia first pass through a priming phase, Ho-Mg, also referred to as CL0/CL1, then a phagocytic phase (CL2), identified by the expression of TREM2, AXL, and APOE, and finally reach an APC-competent phase (CL3 Mg-TAMs). Although this profile suggests a pro-inflammatory programme, its functional relevance is tightly linked to the spatial context and to the metabolic state of the tumour niche in which it occurs.

The spatial distribution of these populations reflects the metabolic architecture of GBM, although studies report partly conflicting data. According to Yabo et al. (2), CL3 Mg-TAM signatures are preferentially localised in reactive immune niches at the tumour periphery and invasive margin, more oxygenated areas that provide the metabolic conditions required to sustain STAT1 activity, whereas the tumour core, characterised by severe hypoxia, lactate accumulation, and suppressive cytokines (IL-10, TGF-β), suppresses STAT1-driven programmes. In contrast, Schmassmann et al. (4) reported downregulation of interferon-response genes (IFI6, STAT1, ISG15) specifically in microglia from the periphery compared with the centre, suggesting that the tumour core may instead contain a more responsive microglial pool, possibly stimulated by infiltrating CD8^+^ T cells (4). Hypoxia-TAMs further remodel the vascular compartment: a subset of monocyte-derived TAMs localised to the peri-necrotic niche destabilises endothelial adherens junctions via adrenomedullin paracrine signalling, promoting hyperpermeable neovasculature that impairs drug delivery; genetic or pharmacological blockade of adrenomedullin restores vascular integrity and improves intratumoral drug concentration (34). Several factors may account for this discrepancy. First, “periphery” and “invasive margin” are not always operationalised identically across studies, and differences in sampling region definitions could capture distinct anatomical compartments. Second, the two studies differ analytically: Yabo et al. relied on signature projection of a mouse-derived programme onto spatial data, whereas Schmassmann et al. performed direct differential expression on human tissue, an approach less susceptible to cross-species signature bias. Third, model system differences, PDOX xenografts versus patient-derived tissue, may themselves shape the local immune architecture. Finally, these findings need not be mutually exclusive: IFN-responsive microglia may redistribute dynamically between core and periphery over the course of tumour progression, depending on local CD8^+^ T-cell infiltration and tumour genotype, such that cross-sectional sampling at different disease stages could capture opposite snapshots of the same dynamic process.

Despite their antigen-presenting potential, the ability of CL3 Mg-TAMs to prime T cells appears constrained by co-expressed inhibitory signals. A key player in this context is osteopontin (SPP1): microglia in “reactive” niches secrete high levels of SPP1, which interacts with CD44 on T and NK cells; this SPP1–CD44 interaction acts as an immune checkpoint that suppresses lymphocyte activation and proliferation, promoting immune evasion (4). Independently, Ravi et al. in a paper published in Nature Communications identified a distinct HMOX1^+^CD163^+^ myeloid population that preferentially localises within mesenchymal-like (MES-like) tumour regions and drives T-cell exhaustion via IL-10 secretion (31). Yabo et al. (2) similarly report that CL3 Mg-TAM signatures co-localise with MES-like tumour cells; taken together, these two independent findings, from different datasets, species compositions, and clustering approaches, raise the possibility that APC-competent microglial states and IL-10/SPP1-mediated suppression converge within the same MES-like regional niche, though this spatial convergence has not been demonstrated within a single dataset and should be treated as a cross-study inference rather than an established co-localisation. It also remains unresolved whether the HMOX1^+^CD163^+^ population of Ravi et al. corresponds, in whole or in part, to any Mg-TAM cluster in the Yabo taxonomy: notably, CL8 in the CL0–CL8 framework is defined in part by downregulation of CD163 (**Table 1**), so the two populations should not be assumed equivalent without further cross-dataset validation (31). We return to the HMOX1^+^/IL-10 axis in detail in the following section.

Thus, the acquisition of an APC-competent and phagocytic transcriptional programme does not necessarily translate into effective tumour clearance, as multiple inhibitory receptor pathways within the tumour microenvironment functionally restrain these cells. Among these, the CD47–SIRPα “don’t-eat-me” axis represents a major mechanism limiting myeloid-mediated phagocytosis and therefore a promising therapeutic target (35).

## Tumour-imposed metabolic constraints

The acquisition of an APC-competent transcriptional programme by CL3 Mg-TAMs represents the highest immunological potential of tumour-associated microglia. However, this transcriptional blueprint can only be translated into effective anti-tumour immunity if permitted by the metabolic architecture of the local tumour niche. This illustrates a fundamental principle of GBM myeloid biology: a transcriptional–functional uncoupling reflects a gap between molecular identity and functional capacity, where transversal modifiers, such as lipid overload, severe hypoxia, and lactate accumulation, can override even the most differentiated pro-inflammatory programmes (18, 36). This distinction is important: a TAM cell may carry the transcriptional signature of an IFN-responsive or APC-competent state while being metabolically unable to execute the corresponding function, because the local niche renders those programmes biochemically unsustainable (2, 32). Within this framework, three metabolic axes emerge as particularly relevant to understanding how the GBM TME enforces myeloid immunosuppressive states; these are displayed in **Figure 3**.

**Figure 3:**
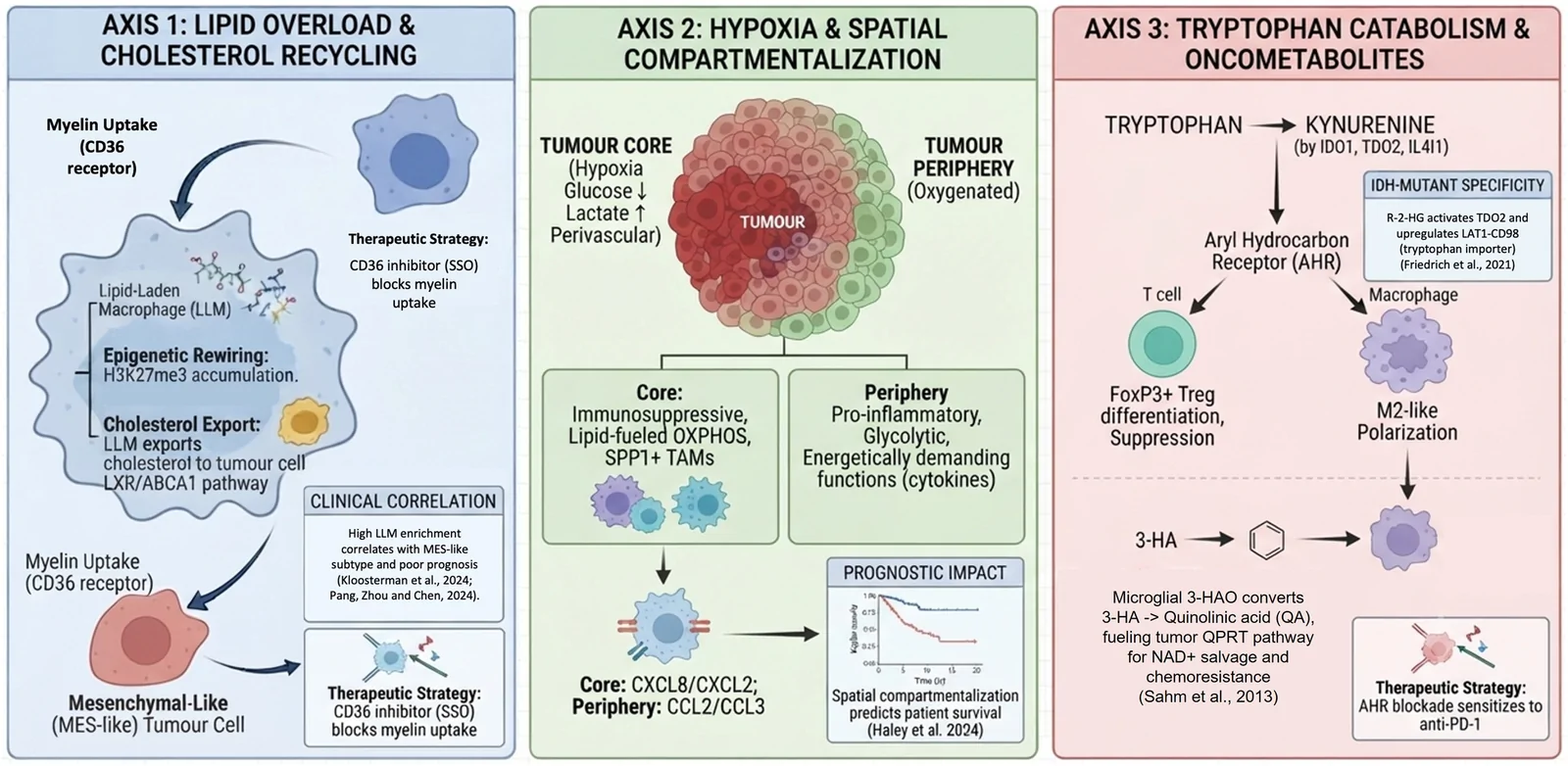
Metabolic constraints shaping TAM function in GBM. Three convergent axes of myeloid functional restriction: (Axis 1) lipid overload and myelin-cholesterol recycling by lipid-laden macrophages via CD36-mediated uptake and LXR/ABCA1-dependent export to mesenchymal-like tumour cells, associated with poor prognosis (32, 39); (Axis 2) hypoxia-driven spatial compartmentalization, with immunosuppressive, lipid-fuelled programmes enriched in the tumour core and pro-inflammatory, energetically demanding programmes at the periphery, predicting patient survival (18); (Axis 3) tryptophan catabolism via IDO1/TDO2/IL4I1 and kynurenine-driven AHR activation, amplified in IDH-mutant tumours by R-2-HG (42), with a parallel microglial 3-HAO/quinolinic acid/QPRT branch sustaining tumour NAD^+^ salvage and chemoresistance (44).

The first axis of functional restriction in GBM is lipid overload, a phenomenon initially identified through the presence of GPNMB-high macrophages (37) and recently mechanistically defined as lipid-laden macrophages (LLMs) by Kloosterman et al. (32). These cells establish a potent metabolic symbiosis with aggressive MES-like tumour cells: LLMs actively scavenge cholesterol-rich myelin debris from the tumour microenvironment via the CD36 receptor and, rather than degrading these lipids, export them to the tumour in an LXR/ABCA1-dependent manner. This recycling circuit not only provides essential building blocks for tumour proliferation and membrane construction but also shields cancer cells from the lipotoxic effects of raw myelin debris, which would otherwise diminish their viability (32).

In contrast to earlier models that focused on simple fatty acid oxidation dysfunction, recent evidence demonstrates that myelin internalisation triggers a profound epigenetic rewiring. The accumulation of myelin-derived cholesterol leads to an increase in the repressive histone mark H3K27me3, driven by the upregulation of EZH2 and the loss of the demethylase KDM6B, which actively silences pro-inflammatory and antigen-presenting genes (32). Beyond this epigenetic mechanism, TREM2^+^ microglia-derived macrophages more broadly display a dual immunosuppressive and lipid-metabolic phenotype, with enrichment of fatty acid metabolism and lipoprotein clearance genes, including marked upregulation of the TREM2 ligand APOE. Clinically, high TREM2 expression correlates with elevated tumour grade, recurrence, and shorter overall and disease-free survival, positioning TREM2 as a robust prognostic marker; the prognostic association of APOE itself, by contrast, is less consistent across cohorts (38). This lipid-laden signature shares substantial marker overlap with the TAM-SPP1 phenotype described by Tang et al. (16), frequently enriched in tumours harbouring EGFR amplification and driving T-cell exhaustion through the SPP1–CD44 and LGALS9–CD44 signalling axes, although, as noted above, the TAM-SPP1 population itself was defined in grade 2–3 glioma tissue rather than GBM; the two populations should therefore be regarded as closely related on transcriptional grounds rather than formally established as the same entity across tumour grades.

The clinical significance of this metabolic axis is underscored by the finding, from Kloosterman et al. (32) itself, that high LLM enrichment correlates with the aggressive mesenchymal subtype and poor patient prognosis, an association subsequently highlighted in a commentary on this same study (39). Consequently, pharmacological disruption of this axis, for example, using the blood–brain barrier-permeable CD36 inhibitor sulfosuccinimidyl oleate (SSO) to block myelin uptake, represents a promising therapeutic strategy to sensitise tumours to radiotherapy and counteract resistance to immune checkpoint blockade, based on the survival benefit reported in the PDG-Ink4a mouse model (32).

The second axis of functional restriction in GBM is hypoxia, which drives the spatial compartmentalisation of myeloid populations in GBM: multimodal mapping demonstrates that distinct myeloid subsets occupy reproducible spatial niches defined by hypoxia gradients, regional chemokine signalling (e.g., CXCL8/CXCL2 in the core vs. CCL2/CCL3 in the periphery), and heterotypic cellular interactions, and that these spatial patterns predict patient survival (18). At the hypoxic tumour core, where glucose is limiting and lactate accumulation is maximal, myeloid cells are preferentially enriched in immunosuppressive and lipid-accumulating programmes often associated with the perivascular niche and BBB breakdown. This spatial gradient directly constrains microglial functional programmes: transcriptional states requiring energetically demanding outputs, such as cytokine secretion, are restricted to niches where metabolic substrates are available, while the tumour core enforces lipid-fuelled, OXPHOS-dependent, immunosuppressive states consistent with the lipid-laden TAM phenotype described above.

The third axis of functional restriction in GBM is tryptophan catabolism, which represents a primary metabolic mechanism of immune evasion, mediated by the rate-limiting enzymes IDO1, TDO2, and IL4I1. These enzymes catalyse the conversion of tryptophan into kynurenine, a potent endogenous ligand for the aryl hydrocarbon receptor (AHR), a molecular switch that, once activated, “freezes” the immune response (40). In a pan-cancer analysis, IL4I1 was found to associate more strongly with AHR activity than IDO1 or TDO2; importantly, IL4I1 is not suppressed by conventional IDO1 inhibitors, a mechanistic gap that likely contributed to the failure of clinical trials targeting IDO1 (41). In IDH-mutant high-grade gliomas, this pathway is further amplified by the oncometabolite R-2-HG, which establishes an intrinsic immunosuppressive loop: R-2-HG acts as an allosteric activator of TDO2 in monocyte-derived macrophages, while concurrently triggering an amino acid starvation-like response that upregulates the tryptophan importer LAT1–CD98 (SLC7A5/SLC3A2) (42). This metabolic rewiring sustains kynurenine production even in substrate-limited environments. Kynurenine-mediated AHR activation further drives a Treg–macrophage suppressive axis, promoting FoxP3^+^ Treg differentiation and M2-like polarisation of TAMs (40, 43). Pharmacological AHR blockade has been shown to counteract this axis, synergising with PD-1/PD-L1 inhibition to enhance anti-tumour T-cell responses and prolong survival in IDH-mutant models (40, 42). Notably, this AHR-amplifying effect of R-2-HG operates in parallel with, and is mechanistically distinct from, its epigenetic suppression of chemokine expression discussed later in this review (see “Therapeutic opportunities and limitations”): the two should not be read as contradictory, since one reflects direct allosteric enzyme activation while the other reflects promoter hypermethylation of a separate gene set.

Beyond AHR activation, a specialised branch of this pathway exists at the intersection of microglial metabolism and tumour resistance. In recurrent GBM, microglia-specific 3-hydroxyanthranilic acid oxygenase (3-HAO) converts tryptophan-derived 3-HA into quinolinic acid (44). This metabolite is subsequently utilised by glioma cells via the QPRT-mediated NAD^+^ *de novo* pathway, which provides the metabolic substrates necessary for DNA repair and resistance to oxidative stress induced by radiotherapy and alkylating agents (44). Thus, tryptophan metabolism not only suppresses adaptive immunity but directly fuels the tumour’s chemoresistance machinery through microglial cooperation.

## Immunosuppressive circuits in the GBM microenvironment

The metabolic constraints previously described, lipid overload, hypoxia, and tryptophan catabolism, do not merely act as passive barriers to anti-tumour immunity; rather, they actively promote the establishment of self-sustaining immunosuppressive circuits. These circuits represent a further layer through which transcriptional–functional uncoupling is enforced: cells retaining pro-inflammatory or APC-competent transcriptional identity are actively silenced at the signalling level. A central node within these networks is the production of interleukin-10 (IL-10), a potent immunoregulatory cytokine that orchestrates T-cell dysfunction. While multiple pathways converge on IL-10 induction, including activation of the tryptophan-derived kynurenine/AHR axis, Ravi et al. used an integrative single-cell and spatial transcriptomic approach across 45,615 immune cells from 12 GBM patients to identify a discrete HMOX1^+^CD163^+^ myeloid subset as the principal source of IL-10 within the tumour microenvironment (31). The identity of these cells is defined by high co-expression of heme oxygenase-1 (HMOX1), an enzyme induced by oxidative stress and metabolic imbalance, and the scavenger receptor CD163. Importantly, these cells are not randomly distributed but preferentially localise within mesenchymal-like tumour regions, where they co-localise with T cells displaying advanced exhaustion signatures (31). This IL-10-driven mechanism was subsequently tested functionally in an ex vivo human neocortical GBM tissue-slice model inoculated with patient-derived T cells to simulate the lymphoid compartment; in this system, pre-treatment with ruxolitinib, a JAK1/2 inhibitor, reduced IL-10 signalling and partially restored T-cell effector activity, supporting a causal, rather than purely correlative, role for the HMOX1^+^CD163^+^–IL-10 axis in driving T-cell dysfunction within this model. On the basis of these ex vivo findings, the same group reported a single recurrent GBM patient treated with neoadjuvant ruxolitinib as an exploratory case study; while consistent with the proposed mechanism, this remains a single-patient observation rather than clinical trial evidence and should be interpreted accordingly when considering IL-10/JAK-STAT blockade as a therapeutic strategy (discussed further below). Collectively, these findings identify the HMOX1^+^CD163^+^–IL-10 axis as a major downstream effector through which the metabolically reprogrammed GBM microenvironment reinforces immune suppression (31). Additional macrophage subsets contribute to tumour progression through mechanisms distinct from the IL-10 axis. GPNMB-high macrophages have been shown to promote proneural-to-mesenchymal (PN–MES) transition while simultaneously impairing T-cell activation, identifying GPNMB as a potential therapeutic target (37). Two further immunosuppressive circuits, identified in 2025, extend this landscape. First, an integrative analysis combining bulk RNA-seq, scRNA-seq, and spatial transcriptomics identified VSIG4^+^S100A10^+^ TAMs as a distinct immunosuppressive subset in which VSIG4 drives M2-like polarisation and tumour progression; spatial and multiplex immunofluorescence data confirmed co-localisation of this population with multiple T-cell types and suppression of their cytotoxic function, with VSIG4^+^S100A10^+^ TAM abundance serving as an independent poor-prognosis indicator and being enriched in anti-PD-1 non-responders (45). Second, LILRB4, an inhibitory immunoglobulin-like receptor predominantly expressed in M2-like TAMs, was identified as an independent prognostic factor in glioma; single-cell and spatial data localise LILRB4 to macrophage clusters co-occurring with tumour cells, and its knockdown shifts macrophages toward an M1-like phenotype via downregulation of *CD163, IL10* and *TGFB1* and upregulation of *IL1B* and *TNF*, acting through a STAT3/IL10 axis upstream of, or parallel to, the HMOX1^+^CD163^+^ circuit described above (46).

### Myeloid derived suppressor cell subtypes: early-stage, monocytic, and polymorphonuclear

Unlike the CL0–CL8 taxonomy, which describes states along the microglial (brain-resident) lineage, myeloid derived suppressor cells (MDSCs) are ontogenetically distinct: they originate from bone marrow myelopoiesis and infiltrate the tumour as immature peripheral cells rather than arising through *in situ* reprogramming of resident microglia. MDSC subsets should therefore be regarded as largely orthogonal to, rather than a subset of, the CL0–CL8 framework, and their relationship to lipid-laden Mo-TAMs and the HMOX1^+^CD163^+^ population remains to be resolved through direct cross-dataset comparison. This ontogenetic distinction underscores that effective myeloid-targeting strategies must account separately for brain-resident (microglia-derived) and peripheral (bone marrow-derived) lineages. Jackson et al. identified two additional bone marrow-derived populations, E-MDSC (early-Myeloid-Derived Suppressor Cell) and M(Monocytic)-MDSC, that were enriched in IDH-wildtype glioblastoma; in IDH-mutant gliomas, these cells were reduced in parallel with broader epigenetic repression of chemotactic and immune-recruiting programs (47, 48). The suppressive activity of these MDSCs is sustained by a robust metabolic-hypoxic program, with E-MDSCs preferentially localizing to pseudopalisading regions where they engage in reciprocal crosstalk with stem-like tumour cells. In this symbiotic niche, E-MDSCs produce growth factors, notably FGF11 (interacting with FGFR1) and VCAN (interacting with EGFR), to promote tumour aggressiveness. Whether the mechanisms underlying MDSC-mediated suppression are non-redundant with respect to the IL-10/JAK-STAT axis operative in HMOX1^+^CD163^+^ cells remain an open question, as current spatial datasets do not yet permit definitive mechanistic conclusions on their topographical overlap. These findings underscore that effective myeloid-targeting strategies must account for multiple, potentially parallel immunosuppressive axes.

Importantly, the IL-10-centered axis does not exhaust the immunosuppressive myeloid landscape of IDH-wildtype GBM. In human GBM, circulating and tumour-infiltrating MDSCs are heterogeneous and frequently dominated by a granulocytic/neutrophil-like PMN-MDSC fraction (CD15^+^CD33^+^HLA-DR^−^), which in several cohorts accounted for the majority of peripheral MDSCs and contributed to arginase-associated T-cell (49, 50). Analyses of resected human GBM also confirmed MDSC infiltration *in situ*, with granulocytic, lineage-negative, and monocytic subsets co-existing within the tumour and the granulocytic fraction remaining biologically relevant across specimens (49). More recent work identifies LOX-1 as a selective marker for human PMN-MDSCs and shows that LOX-1^+^ PMNs in GBM are enriched in tumour tissue, express ARG1/iNOS and high ROS, inhibit CD3^+^ T-cell proliferation, and correlate with worse clinical features, supporting a bona fide immunosuppressive PMN-MDSC compartment in patients (51). Importantly, PMN-MDSCs are often difficult to capture and annotate consistently across profiling platforms, so orthogonal validation by flow cytometry and immunofluorescence remains essential when interpreting single-cell atlases of the GBM myeloid compartment.

### Role of extracellular vesicles

Beyond transcriptional reprogramming driven by diffusible TME cues, GBM cells actively reshape the microglial compartment through the delivery of instructional cargo via extracellular vesicles (EVs). Using an *in vivo* model of miR-21-null recipient mice, Abels et al. (52) provided direct causal evidence that GBM-derived EVs expand tumour-supportive microglial populations. The functional delivery of miR-21 results in the downregulation of the anti-proliferative target Btg2, triggering a significant increase in Ki67^+^ microglial proliferation (52). Interestingly, while both microglia and infiltrating macrophages internalize tumour EVs, the functional transfer of miRNA cargo appears more efficient in resident microglia, potentially due to specialized uptake mechanisms such as macropinocytosis. In GBM-derived EVs, miR-21 is frequently co-transferred with miR-451, both of which target the c-Myc mRNA. The resulting downregulation of c-Myc in recipient microglia facilitates a phenotypic shift toward an activated, immunosuppressive state characterized by increased Arginase-1 (Arg-1) expression and a pro-tumorigenic cytokine profile (e.g., upregulation of IL-6, CCL2, and CCL5) (53). Furthermore, miR-27a-3p has been identified as a critical EV component that drives M2-like polarization. Mechanistically, EV-delivered miR-27a-3p suppresses the histone methyltransferase EZH1, which normally restrains M2 polarization. The loss of EZH1 allows for the KDM3A-mediated upregulation of Connective Tissue Growth Factor (CTGF), further aggravating tumour invasion and immune evasion (54). Importantly, this EV-mediated crosstalk is increasingly recognized as a bidirectional axis. Microglia-derived small EVs (sEVs) enriched with miR-124 have been shown to rewire glioma metabolism, suppressing lactate and glutamate release, which in turn reduces tumour growth and prolongs survival in preclinical models (55). Given the combinatorial nature of EV cargo, therapeutic strategies targeting global EV biogenesis or uptake, such as the use of heparin-like competitive inhibitors, may prove more effective than interventions targeting single miRNAs (53).

### Soluble factors

In parallel to vesicle-mediated communication, GBM cells secrete soluble factors that reprogram resident microglia and recruit bone marrow-derived macrophages (BMDMs) through distinct receptor-mediated mechanisms. GBM cells overexpress CSF1 and its functional paralogue IL-34, which engage CSF1R on myeloid cells to sustain their survival and pro-tumoral polarisation. While CSF1R inhibition (e.g., BLZ945, PLX3397) depletes TAMs in the healthy parenchyma, TAMs within the tumour mass are often preserved due to protective signals such as GM-CSF and IFN-γ. In this context, CSF1R blockade elicits a potent anti-tumour response not through physical depletion, but by “re-educating” TAMs toward a highly phagocytic, M2-low phenotype (56). Importantly, this study did not resolve microglia and macrophages as separate ontogenetic populations. These observations support an important concept emerging from recent studies: immunosuppressive myeloid circuits are not irreversibly fixed but retain a considerable degree of functional plasticity. Rather than eliminating TAMs, CSF1R inhibition demonstrates that therapeutic reprogramming toward a less immunosuppressive, more phagocytic phenotype is achievable, even within the tumour microenvironment (56). However, the myeloid compartment also displays remarkable adaptive capacity under therapeutic pressure. Acquired resistance to CSF1R blockade is driven by a tumour microenvironment-initiated paracrine loop in which surviving TAMs secrete IGF-1, activating IGF-1R and the PI3K/AKT pathway in glioma cells and ultimately promoting tumour rebound. Combined inhibition of CSF1R and IGF-1R overcomes this adaptive resistance and significantly prolongs survival in preclinical models (57). A second adaptive axis involves CCL2/CCR2 signalling, which mediates the recruitment of inflammatory monocytes from the periphery. Pharmacological disruption of this pathway prevents the egress of immunosuppressive MDSCs from the bone marrow, reduces tumour infiltration, and effectively unmasks the efficacy of PD-1 checkpoint blockade (58). The convergence of these compensatory pathways on shared signalling hubs, including STAT3 and NF-κB, further underscores the need for combinatorial therapeutic strategies rather than single-agent myeloid target.

### Temozolomide treatment

Myeloid states in GBM are not static but are continuously re-educated by the standard-of-care. Yabo et al. (2) demonstrated that TMZ treatment induces significant transcriptomic plasticity, triggering an inflammatory rewiring of Mg-TAMs and activating pro-phagocytic “eat-me” pathways like Gas6. Furthermore, post-treatment recurrence is often characterized by an ontogeny switch, where monocyte-derived TAMs outcompete resident microglia, particularly within the hypoxic and necrotic niches exacerbated by therapy-induced vascular damage. These dynamics argue for temporal sequencing of myeloid-targeting interventions, potentially following cytoreductive surgery or during the “nadir” periods after chemotherapy, to exploit windows of reduced immunosuppressive reprogramming.

## Therapeutic opportunities and limitations

The therapeutic landscape for GBM is undergoing a fundamental transition from broad myeloid cell depletion toward lineage-aware and niche-specific modulation. The individual strategies below are summarized in **Figure 4** and mapped onto their core mechanistic themes in **Table 3**.

**Figure 4:**
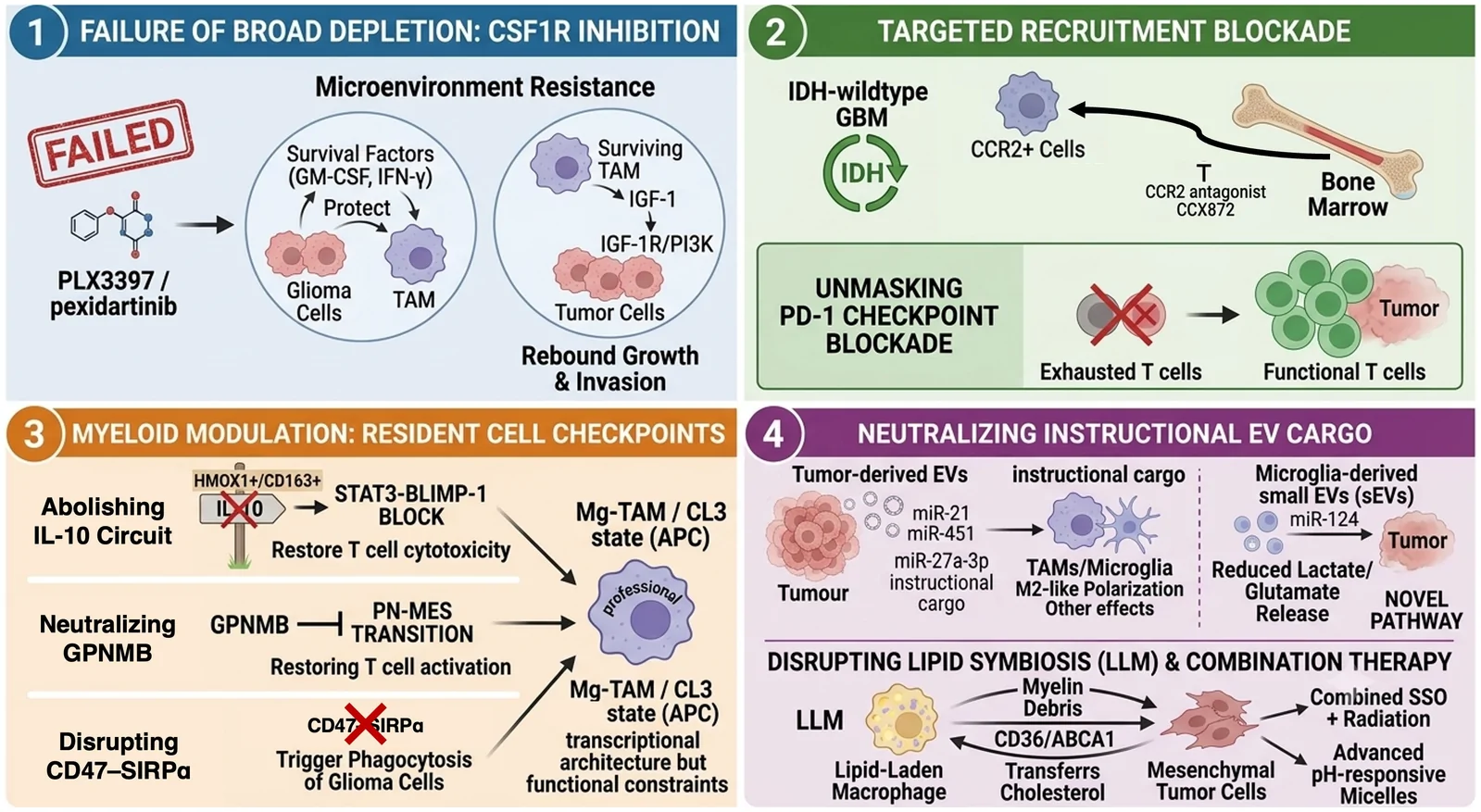
Strategies targeting the myeloid compartment in glioblastoma. (1) CSF1R inhibition (PLX3397/pexidartinib) fails clinically due to microenvironment-driven resistance, in which glioma-derived survival factors (GM-CSF, IFN-γ) protect TAMs, and surviving TAMs secrete IGF-1 to drive tumour rebound via IGF-1R/PI3K signalling (56, 57, 59). (2) In IDH-wildtype GBM, CCR2 blockade (CCX872) prevents egress of CCR2^+^ MDSCs from the bone marrow to the tumour, reducing infiltration and unmasking the efficacy of PD-1 checkpoint blockade (58). (3) Reprogramming CL3 Mg-TAMs toward productive antitumour function by targeting co-occurring inhibitory checkpoints — the IL-10/STAT3–BLIMP-1 axis, GPNMB, and CD47–SIRPα, to restore T-cell activation and myeloid-mediated phagocytosis (4, 31, 37). (4) Disrupting tumour–myeloid crosstalk by neutralizing instructional extracellular vesicle cargo (52, 53, 55) and blocking lipid-laden macrophage–mesenchymal cell metabolic symbiosis via CD36/ABCA1 inhibition, combined with radiotherapy or advanced delivery platforms (32, 67).

**Table 3:** Therapeutic targets in GBM myeloid reprogramming, mapped onto core mechanistic themes.

Target/intervention	Core mechanistic axis	IDH relevance	Reference
CSF1R inhibition (PLX3397 ± piperlongumine/vorinostat)	Recruitment/depletion; metabolic resistance mechanism	Not stratified	Pyonteck et al., 2013 (56); Quail et al., 2016 (57); Butowski et al., 2016 (59); Miao et al., 2025 (60)
CCR2 blockade (CCX872)	Recruitment (MDSC egress)	IDH-wildtype*	Flores-Toro et al., 2020 (58)
Demethylation/R-2-HG pathway inhibition	Epigenetic reprogramming	IDH-mutant	Amankulor et al., 2017 (48); Luoto et al., 2018 (61)
AHR blockade	Metabolic symbiosis (tryptophan axis)	IDH-mutant (amplified)	Campesato et al., 2020 (40); Friedrich et al., 2021 (42)
JAK1/2 inhibition (ruxolitinib)	Receptor-mediated checkpoint	Not stratified	Ravi et al., 2022 (31)
GPNMB neutralization	Receptor-mediated checkpoint	Not stratified	Xiong et al., 2022 (37)
CD47–SIRPα blockade	Receptor-mediated checkpoint (uncoupling)	Not stratified	Li et al., 2018 (35)
LAPTM4A targeting	Intracellular reprogramming	Not stratified	Geng et al., 2025 (64)
KDM6B deletion	Epigenetic reprogramming	Not stratified	Goswami et al., 2023 (66)
CD36/ABCA1 blockade (SSO)	Metabolic symbiosis (lipid axis)	Not stratified	Kloosterman et al., 2024 (32)
EV/miR-124 modulation	Intercellular communication	Not stratified	Abels et al., 2019 (52); Serpe et al., 2021 (55)
TMZ-induced window + delivery platforms	Temporal/combinatorial sequencing	Not stratified	Yabo et al., 2024 (2); Li et al., 2024 (67)

Twelve strategies discussed in this review, organized by the mechanistic axis they primarily address and by IDH-status relevance as reported in the cited primary literature, except where noted. *Inferred from the GL261 model system; not stated as an explicit IDH-status stratification in the original study.

### Recruitment and depletion strategies

The clinical experience with CSF1R inhibition provides a critical cautionary lesson; the small molecule PLX3397 (pexidartinib) failed to achieve efficacy in Phase II trials for both recurrent and newly diagnosed GBM (59). This failure is primarily rooted in microenvironment-driven resistance, where glioma cells secrete survival factors like GM-CSF and IFN-γ that protect TAMs from apoptosis (56). Furthermore, sustained blockade triggers a paracrine loop in which surviving TAMs secrete IGF-1, activating the IGF-1R/PI3K pathway in tumour cells to fuel rebound growth and invasion even under treatment (57). More recent work has moved from monotherapy toward combination strategies to overcome this resistance. Translatome profiling (TRAP-seq) identified oxidative phosphorylation/glycolysis metabolic reprogramming as a key driver of anti-CSF1R resistance and showed that piperlongumine or vorinostat could reverse this metabolic rewiring and restore PLX3397 sensitivity; a triple combination of PLX3397, temozolomide, and piperlongumine/vorinostat significantly prolonged survival in GBM mouse models (**Figure 4**) (60).

A more targeted approach focuses on disrupting the recruitment of highly immunosuppressive myeloid-derived suppressor cells (MDSCs). The CCL2/CCR2 axis is essential for this process, particularly in IDH-wildtype GBM, where tumour-derived CCL2 (and CCL7) recruits CCR2^+^ cells from the bone marrow to the tumour microenvironment (58). Pharmacological inhibition of this axis using the CCR2 antagonist CCX872 prevents MDSC egress from the bone marrow, effectively reducing tumour infiltration (58). Notably, CCR2 inhibition has been shown to unmask the efficacy of PD-1 checkpoint blockade in resistant models, significantly extending overall survival by increasing the infiltration of functional, non-exhausted T cells (58). This CCL2/CCR2-driven myeloid recruitment is a hallmark of IDH-wildtype GBM; conversely, in IDH-mutant gliomas, R-2-HG suppresses a broader immunosuppressive/chemotactic transcriptional programme rather than promoting it. Using isogenic murine glioma models, Amankulor et al. demonstrated that R-2-HG-associated global CpG island hypermethylation coincides with reduced expression of multiple chemokines and cytokines (including CCL-2, CCL-3, CXCL-1, CXCL-2, CXCL-16, GM-CSF, IL-2, and IL-6) resulting in impaired leukocyte and neutrophil chemotaxis and reduced immune cell infiltration into the tumour (48). This is consistent with broader evidence that IDH1-mutant, G-CIMP-positive GBM samples show lower expression and higher promoter DNA methylation of immune-related genes such as MHC-I (HLA-A/B/C), with expression restored by pharmacological demethylation in IDH-mutant glioma cells (61). Taken together, these findings indicate that IDH mutation status is not a peripheral variable but a primary determinant of the GBM myeloid landscape. IDH-wildtype GBM is characterised by robust CCL2/CCR2-driven recruitment of peripheral MDSCs, active tryptophan–kynurenine–AHR signalling, and a myeloid compartment amenable to CCR2 blockade and checkpoint combination strategies. IDH-mutant gliomas, by contrast, show a globally suppressed chemotactic and antigen-presentation programme driven by R-2-HG-mediated CpG hypermethylation, resulting in reduced myeloid infiltration but also reduced responsiveness to recruitment-blocking strategies; here, pharmacological demethylation or direct R-2-HG pathway inhibition may be more rational entry points than CCR2- or CSF1R-targeted approaches. Large-scale atlases such as GBmap, which specifically profiled IDH-wildtype GBM across 240 patients, provide an important reference for benchmarking these wildtype-specific myeloid programmes and underscore the need for analogous IDH-mutant-specific single-cell resources (**Figure 4**) (30). Emerging multi-omic evidence also implicates the gut microbiota in shaping GBM immune, RNA-methylation and metabolic programmes, adding a further layer of host-level variability to myeloid reprogramming (62).

### Reversing transcriptional–functional uncoupling: receptor-mediated checkpoints

Restoring the anti-tumour potential of resident cells involves modulating the CL3 Mg-TAM state described in Yabo et al. (2). While these microglia-derived cells carry the transcriptional architecture of professional APC-like cells, including MHC-II and CD86, they are functionally constrained by co-expressed inhibitory signals (2). This antigen-processing/presentation programme is consistent with a pan-cancer single-cell atlas identifying IFI30^+^ macrophages as key modulators of intra-tumoral antigen-processing and immune dynamics across tumour types, suggesting that the CL3-associated MHC-II machinery described here may reflect a broader, cross-cancer myeloid programme rather than a GBM-specific phenomenon (63).

Three checkpoints arise as primary targets. First, the pharmacological inhibition of JAK1/2 signalling with ruxolitinib attenuated the downstream response to IL-10 and partially restored T-cell effector activity. These findings indicate that IL-10-mediated signalling contributes functionally to T-cell dysfunction in this model, rather than merely correlating with it, while also suggesting that additional immunosuppressive mechanisms may remain active (31). Second, neutralizing GPNMB represents a novel strategy to block the macrophage-driven proneural-to-mesenchymal (PN-MES) transition while restoring T cell activation (37). Third, disrupting the CD47–SIRPα “don’t-eat-me” signal restores myeloid-mediated tumour clearance by promoting the phagocytosis of both glioma cells and glioma stem cells (GSCs) (35). This strategy is particularly attractive in the context of CL3 Mg-TAMs, as it may unleash the phagocytic potential of a population that is already transcriptionally primed for tumour recognition and antigen presentation. An additional reprogramming target is LAPTM4A, whose loss in glioma models shifts TAMs toward an M1-like phenotype, enhances immune activation, and sensitises tumours to anti-PD-1 therapy, identifying LAPTM4A as a candidate node for pharmacological TAM reprogramming (**Figure 4**) (64).

### Converging on shared signalling nodes

These findings collectively point to a small set of convergent signalling hubs, STAT1/STAT3, NF-κB, PI3K/AKT, and HIF-1α, that recur across otherwise distinct myeloid states and regulatory axes discussed throughout this review. The STAT1-driven regulon underlying the CL3 APC-competent programme (2), the LILRB4-STAT3/IL10 axis (46), and the HMOX1^+^CD163^+^-IL-10/JAK-STAT3 circuit all converge on STAT-family signalling, while HIF-1α links hypoxia-driven metabolic reprogramming (Axis 2, **Figure 3**) to downstream NF-κB and PI3K/AKT activity. This convergence extends to tumour-myeloid crosstalk itself: macrophage- and microglia-derived oncostatin M (OSM) activates STAT3 signalling through OSMR/LIFR–GP130 complexes on GBM cells, driving proneural-to-mesenchymal transition, which in turn reinforces a pro-tumorigenic macrophage phenotype in a reciprocal feed-forward loop (65). Rather than acting as independent pathways, STAT3, NF-κB, PI3K/AKT and HIF-1α therefore form an interconnected regulatory network whose convergence may explain why single-node interventions (e.g., CSF1R or JAK1/2 inhibition alone) have shown limited clinical efficacy, and why combinatorial strategies targeting multiple nodes simultaneously may be required. Epigenetic reprogramming of myeloid cells represents an additional therapeutic avenue: myeloid-specific deletion of KDM6B, a H3K27me3 demethylase, enhances antigen presentation, interferon response and phagocytosis by suppressing Mafb, SOCS3 and Sirpa, improving survival and anti-PD1 efficacy in GBM models (66).

### Metabolic symbiosis and intercellular communication

Beyond targeting established immunosuppressive circuits, an alternative therapeutic strategy is to prevent the continuous tumour-driven reprogramming of the myeloid compartment. One of the principal mechanisms underlying this dynamic crosstalk is the transfer of instructional cargo through tumour-derived extracellular vesicles (EVs) (52). Importantly, this communication is bidirectional: microglia-derived small EVs (sEVs) containing miR-124 can reprogramme glioma metabolism by reducing lactate and glutamate release, highlighting the therapeutic potential of manipulating EV-mediated signalling (**Figure 4**) (55).

Finally, strategies must account for the metabolic symbiosis established between the tumour and aggressive LLMs. These characteristically GPNMB-high cells establish a transport hub by scavenging cholesterol-rich myelin debris and transferring these lipids to mesenchymal tumour cells in an ABCA1/LXR-dependent manner (32). Disruption of this axis via CD36 inhibition (using BBB-permeable such as sulfosuccinimidyl oleate SSO) or ABCA1 blockade curbs mesenchymal growth and sensitizes the tumour to radiotherapy (32). Combined with TMZ, which induces a temporary window of pro-inflammatory and APC-like rewiring in Mg-TAMs, and advanced delivery platforms like pH-responsive hierarchical micelleplexes, these niche-specific interventions offer a path toward overcoming the historical failures of myeloid-targeted therapy in GBM (**Figure 4**) (67).

## Conclusions

The traditional framing of myeloid activation as a binary M1/M2 polarization model has been fundamentally challenged by recent single-cell and spatial profiling studies (2, 4, 14, 68). Crucially, the tumour’s genetic drivers strictly dictate these instructional cues; for instance, the recruitment of early myeloid-derived suppressor cells (E-MDSCs) and monocytic MDSCs (M-MDSCs) is a hallmark of aggressive IDH-wildtype GBM, whereas these populations are virtually absent in IDH-mutant gliomas due to the epigenetic silencing of MDSC-attracting chemokines via the oncometabolite R-2-HG (42, 47).

The transcriptional–functional uncoupling introduced at the outset of this review is the central concept emerging from recent single-cell studies: transcriptional activation does not necessarily translate into effective immune function. This principle finds its clearest example in the CL3 Mg-TAM state described by Yabo et al. in which microglia acquire an APC-like/IFN-responsive transcriptional program but available data suggest that they remain functionally constrained within the tumour microenvironment. Rather than representing a fully productive antigen-presenting state, this program appears to be maintained in a non-productive configuration by suppressive receptor-mediated checkpoints, including CD47–SIRPα and SPP1–CD44, together with cytokine circuits such as the HMOX1^+^/IL-10/JAK-STAT axis and broader metabolic restrictions (16, 31, 35). A key limitation underpinning much of this framework is that the CL0–CL8 taxonomy, and in particular the pro-tumorigenic CL3 state central to the therapeutic rationale discussed above, was derived from murine models and has not yet been independently re-clustered and validated in human single-cell data; should future human datasets fail to recapitulate CL3 as defined, the strength of CL3-centred therapeutic strategies would need to be reassessed.

The discovery of LLMs, characterized by a GPNMB-high signature, further emphasizes that myeloid reprogramming is a process of metabolic co-evolution. By hijacking the brain’s homeostatic myelin-recycling machinery via CD36 and ABCA1, LLMs establish a symbiotic transport hub that converts cholesterol-rich myelin debris into a fuel source for aggressive mesenchymal-like tumour cells while simultaneously shielding them from lipotoxicity (32, 39).

Moving forward, the clinical failure of broad myeloid depletion via CSF1R inhibition underscores the inadequacy of “one-size-fits-all” approaches (59). Effective therapeutic strategies must be combinatorial, targeting transcriptional, metabolic, and suppressive programs simultaneously. Furthermore, the efficacy of these interventions is likely dependent on temporal sequencing; the observation that TMZ treatment induces a temporary “window of vulnerability” by shifting Mg-TAMs toward pro-inflammatory and APC-like states suggests that immunotherapies should be timed to exploit these therapy-induced windows of sensitivity (2). Successful translation will also require delivery strategies capable of achieving spatially restricted modulation of the GBM microenvironment such as pH responsive hydrogels (69). Ultimately, a spatially resolved and ontogeny-aware understanding of myeloid biology will be essential for transforming the GBM microenvironment from a sanctuary for tumour growth into a permissive environment for effective anti-tumour immunity.

## Glossary

3-HAO: 3-hydroxyanthranilic acid oxygenase
ABCA1: ATP-binding cassette transporter A1
AHR: aryl hydrocarbon receptor
AIF1: allograft inflammatory factor 1
AKT: protein kinase B
APC: antigen-presenting cell
Arg-1: arginase-1
BBB: blood-brain barrier
BLIMP-1: B lymphocyte-induced maturation protein 1
BMDM: bone marrow-derived macrophage
CCL: C-C motif chemokine ligand (e.g. CCL2, CCL3, CCL4, CCL5, CCL7)
CCR2: C-C chemokine receptor type 2
CCX872: CCR2 antagonist
CD36, CD44, CD47, CD86, CD98, CD163: cluster of differentiation markers
CIITA: class II major histocompatibility complex transactivator
CITE-seq: cellular indexing of transcriptomes and epitopes by sequencing
CLDN5: claudin-5
Clec7a: C-type lectin domain family 7 member A
CNS: central nervous system
CSF1/CSF1R: colony-stimulating factor 1/receptor
CTGF: connective tissue growth factor
CXCL2/CXCL8: C-X-C motif chemokine ligand 2/8
E-MDSC: early-myeloid-derived suppressor cell
EGFR: epidermal growth factor receptor
EV: extracellular vesicle
EZH1/EZH2: enhancer of zeste homolog 1/2
FGF11/FGFR1: fibroblast growth factor 11/receptor 1
FoxP3: forkhead box P3
GBM: glioblastoma
GFAP: glial fibrillary acidic protein
GM-CSF: granulocyte-macrophage colony-stimulating factor
GPNMB: glycoprotein nonmetastatic melanoma protein B
GPR34: G protein-coupled receptor 34
GSC: glioma stem cell
H2-Ab1, H2-Eb1: histocompatibility 2, class II antigen A beta 1 / E beta 1 (murine MHC-II genes)
HLA: human leukocyte antigen
HMOX1: heme oxygenase 1
IDH: isocitrate dehydrogenase
IDO1: indoleamine 2,3-dioxygenase 1
IFI6: IFI27, interferon alpha-inducible protein 6/27
IFN-γ: interferon gamma
IGF-1/IGF-1R: insulin-like growth factor 1/receptor
IL: interleukin (e.g. IL-1B, IL-4, IL-6, IL-10, IL-13, IL-34)
IL4I1: interleukin 4 induced 1
iNOS: inducible nitric oxide synthase
ISG: interferon-stimulated gene
ISG15: interferon-stimulated gene 15
JAK-STAT: Janus kinase–signal transducer and activator of transcription
KDM3A, KDM6B: lysine demethylase 3A/6B
LAT1: L-type amino acid transporter 1 (SLC7A5)
LGALS9: galectin-9
LLM: lipid-laden macrophage
LOX-1: lectin-type oxidized LDL receptor 1
LXR: liver X receptor
M-MDSC: monocytic myeloid-derived suppressor cell
MAF: musculoaponeurotic fibrosarcoma (transcription factor)
MDSC: myeloid-derived suppressor cell
MES: mesenchymal (as in MES-like tumour cell state)
Mg: microglia
Mg-TAM: microglia-derived tumour-associated macrophage
MHC-II: major histocompatibility complex class II
miR: microRNA
MKI67: marker of proliferation Ki-67
Mo-TAM: monocyte-derived tumour-associated macrophage
MX1: MX dynamin-like GTPase 1
NAD+: nicotinamide adenine dinucleotide
NF-κB: nuclear factor kappa B
NR3C1: nuclear receptor subfamily 3 group C member 1 (glucocorticoid receptor)
OPC/NPC: oligodendrocyte precursor cell/neural precursor cell
OSM: oncostatin M
OXPHOS: oxidative phosphorylation
P2RY12: purinergic receptor P2Y12
PD-1/PD-L1: programmed cell death protein 1/ligand 1
PDOX: patient-derived orthotopic xenograft
PECAM1: platelet endothelial cell adhesion molecule 1
PI3K: phosphoinositide 3-kinase
PLX3397: CSF1R inhibitor, pexidartinib
PMN: polymorphonuclear neutrophil
PN-MES: proneural-to-mesenchymal (transition)
QPRT: quinolinate phosphoribosyltransferase
R-2-HG: R-2-hydroxyglutarate
ROS: reactive oxygen species
scRNA-seq: single-cell RNA sequencing
sEV: small extracellular vesicle
SIRPα: signal regulatory protein alpha
SLC3A2: solute carrier family 3 member 2
SOX4: SRY-box transcription factor 4
SPARCL1: SPARC-like protein 1
SPP1: secreted phosphoprotein 1 (osteopontin)
SSO: sulfosuccinimidyl oleate CD36 inhibitor
STAT1/STAT3: signal transducer and activator of transcription 1/3
TAM: tumour-associated macrophage
TCGA: The Cancer Genome Atlas
TDO2: tryptophan 2,3-dioxygenase
TGF-β: transforming growth factor beta
TME: tumour microenvironment
TMEM119: transmembrane protein 119
TMZ: temozolomide
TNF-α: tumour necrosis factor alpha
TOP2A: topoisomerase II alpha
TRAP-seq: translating ribosome affinity purification sequencing
TREM2: triggering receptor expressed on myeloid cells 2
Treg: regulatory T cell
VCAN: versican
